# Green Synthesis of Pristine and Ag-Doped TiO_2_ and Investigation of Their Performance as Photoanodes in Dye-Sensitized Solar Cells

**DOI:** 10.3390/ma16175731

**Published:** 2023-08-22

**Authors:** Abdul Mohshen Sharif, Md. Ashrafuzzaman, Abul Kalam, Abdullah Godran Al-Sehemi, Pankaj Yadav, Brijesh Tripathi, Mrigendra Dubey, Gaohui Du

**Affiliations:** 1 Department of Chemistry, College of Science, King Khalid University, Abha 61413, Saudi Arabia; almohsin831@gmail.com (A.M.S.); ashrafchem568@gmail.com (M.A.); agmasq@gmail.com (A.G.A.-S.); 2 Research Center for Advanced Materials Science (RCAMS), King Khalid University, Abha 61413, Saudi Arabia; 3 Department of Solar Energy, School of Technology, Pandit Deendayal Energy University, Raisan, Gandhinagar 382426, India; pankajphd11@gmail.com; 4 Department of Physics, School of Technology, Pandit Deendayal Energy University, Raisan, Gandhinagar 382426, India; brijesh.tripathi@sse.pdpu.ac.in; 5 Soft Materials Research Laboratory, Discipline of Metallurgy Engineering and Materials Science, Indian Institute of Technology Indore, Simrol, Indore 453552, India; mdubey@iiti.ac.in; 6 Materials Institute of Atomic and Molecular Science, Shaanxi University of Science and Technology, Xi’an 710021, China; dugaohui@sust.edu.cn

**Keywords:** green modified solvothermal synthesis, TiO_2_, Ag-TiO_2_, photovoltaic performance

## Abstract

Dye-sensitized solar cells (DSSCs) have emerged as a potential candidate for third-generation thin film solar energy conversion systems because of their outstanding optoelectronic properties, cost-effectiveness, environmental friendliness, and easy manufacturing process. The electron transport layer is one of the most essential components in DSSCs since it plays a crucial role in the device’s greatest performance. Silver ions as a dopant have drawn attention in DSSC device applications because of their stability under ambient conditions, decreased charge recombination, increased efficient charge transfer, and optical, structural, and electrochemical properties. Because of these concepts, herein, we report the synthesis of pristine TiO_2_ using a novel green modified solvothermal simplistic method. Additionally, the prepared semiconductor nanomaterials, Ag-doped TiO_2_ with percentages of 1, 2, 3, and 4%, were used as photoanodes to enhance the device’s performance. The obtained nanomaterials were characterized using XRD, FTIR, FE-SEM, EDS, and UV–vis techniques. The average crystallite size for pristine TiO_2_ and Ag-doped TiO_2_ with percentages of 1, 2, 3, and 4% was found to be 13 nm by using the highest intensity peaks in the XRD spectra. The Ag-doped TiO_2_ nanomaterials exhibited excellent photovoltaic activity as compared to pristine TiO_2_. The incorporation of Ag could assist in successful charge transport and minimize the charge recombination process. The DSSCs showed a J_sc_ of 8.336 mA/cm^2^, a V_oc_ of 698 mV, and an FF of 0.422 with a power conversion efficiency (PCE) of 2.45% at a Ag concentration of 4% under illumination of 100 mW/cm^2^ power with N719 dye, indicating an important improvement when compared to 2% Ag-doped (PCE of 0.97%) and pristine TiO_2_ (PCE of 0.62%).

## 1. Introduction

The improvement of clean, low-cost, and renewable energy resources is very important for society, and the scientific community is struggling to minimize global warming and satisfy growing energy demands [1,2,3]. In recent years, solar energy has attracted much more attention from the scientific community due to its low cost, CO_2_-free emissions, and clean, environmentally friendly, and easy process [4,5]. More specifically, dye-sensitized solar cells (DSSCs) have drawn considerable interest due to their inexpensive components, including simple fabrication techniques, flexibility, lighter weight, better transparency, higher chemical durability, and clean energy with non-toxic materials when compared to crystalline silicon-based solar cells. Additionally, they have attained promising photovoltaic performance levels in terms of semi-transparency and upscaling for indoor, BIPV, and agrivoltaic applications [6]. Dye-sensitized solar cells (DSSCs) are one of the prospective technologies in third-generation solar cells, which comprise semiconductor photoanodes/dyes/electrolytes/cathodes between glass substrates [7,8]. Photoanodes have been given more focus among these components since they not only transport photo-induced electrons but also serve as a matrix to adsorb dyes, which directly affects the photo-current density. As a result, the semiconductor materials employed as photoanodes play a significant role in the operation of DSSCs [9]. Among various semiconductor metal oxides (Nb_2_O_5_, ZnO, SnO_2_, etc.), anatase TiO_2_ semiconductors are the most promising candidates for high-performance DSSC applications because of their large band gaps (3.2 eV), high chemical stability, low cost, high charge transport capability, biocompatibility, non-toxicity, and electrical properties [10,11,12]. At the moment, the highest reported power conversion efficiency (PCE) in DSSCs is 15.2% with TiO_2_ as a scaffold layer [13].

TiO_2_ has a high recombination rate, which is one of its main problems [8]. Consequently, its high recombination rate has prevented its commercialization, even if it has an appropriate band gap value. It has been shown that these kinds of problems can be solved by surface engineering/passivation with various metal ions [8]. The surface passivation also improves the optoelectronic properties, long-term stability, and the PCE of devices, as well as being able to decrease the density of defect states [14]. Furthermore, for improving the photovoltaic properties of TiO_2_ for DSSC applications, it is essential to increase dye molecule adsorption activity, surface area, charge transfer, and the light harvesting effect of TiO_2_ [15]. The doping approach with noble metals (Ag, Cu, Au, and Pt) has been widely employed to synthesize TiO_2-_based photoelectrode materials for DSSCs in order to improve charge transfer efficiency and optical, structural, electrochemical, and electronic properties [11,16]. Additionally, the doping of these metals enhances the photovoltaic performance of devices because of their faster electron transport time, positive band edge movement, and low electron–hole pair recombination rate [17]. Among these metal ions, silver ions as dopants have drawn more attention in the application of DSSCs because of their low costs, simple preparation methods, and excellent stability under ambient conditions [7]. In previous studies, Ag-doped TiO_2_ has been used as a successful photoanode to improve the power conversion efficiency of DSSCs [11]. Gupta et al. [18] prepared Ag-doped TiO_2_ with different contents of Ag in the range of 1% to 7% by a modified sol–gel method, as well as comparing the performance of undoped and doped TiO_2_ using hexamethylenetetramine (HMT) as a capping agent. The overall power conversion efficiencies of undoped and Ag-doped TiO_2_ (1%Ag) with N719 dye were 0.14% and 0.40%, respectively. Sun et al. synthesized undoped and Ag-doped TiO_2_ nanoparticles/nanofibers with different reaction times of silver content through the electrospinning method. The PCE of doped TiO_2_ nanofibers increased from 5.39% to 6.39%, and compared to those without doping, the PCE of doped photoanodes was enhanced by 18.0% [19]. To prepare the doped TiO_2_ nanoparticles, various methods, such as microwave-derived polyols, the sol–gel process, electrospinning, hydrothermal methods, and green-modified solvothermal methods, have been used. The green-modified solvothermal method is an excellent and cost-effective method among these approaches. Among various techniques, only this technique is used to achieve specific objectives such as controlled crystallite size, high limpidity, minimized agglomeration, and virtuous uniformity [15,20]. Many studies have been reported on pristine and doped TiO_2_ by green synthesis using plant extracts such as Nyctanthes arbortristis, Catharanthus roseus, Eclipta prostrata, and Azadirachta indica for different types of applications [21,22].

In this current study, through the use of a facile modified solvothermal method, we report the green synthesis of pristine and Ag-doped TiO_2_ nanomaterials with a precipitating agent of diethylamine and a capping agent of fig leaf extract for application in a DSSC photoanode. Moreover, the photovoltaic performance of DSSCs’ configuration with the standard dye N719 is reported in this study.

## 2. Materials and Methods

### 2.1. Materials 

Titanium isopropoxide Ti (OCH(CH_3_)_2_))_4_ (99.9%, Aldrich, Kansas City, MO, USA), silver nitrate AgNO_3_ (99%, BDH, Conestoga, PA, USA), ethanol CH_5_OH, acetone (CH_3_)_2_CO, distilled water, and diethylamine ((CH_3_CH_2_)_2_NH) (99% Purity, CheMondis, Cologne, Germany) were used as received.

### 2.2. Preparation of Fig Leaf Extract

The fresh whole fig leaves were collected from the local area of Abha, Asir region, Saudi Arabia, and washed with distilled water. After repeated washes, the whole leaves were allowed to dry at room temperature for 72 h. Next, the dried leaves were completely ground to obtain a powder. After making the powder, 20 g of the sample was added into distilled water (100 mL) at 80 °C with stirring for 24 h to obtain a dark-colored extract. The resultant extract was filtered with filter paper, and subsequently, centrifugation at 4000 rpm for 10 min was employed to eliminate the small dust particles from the solution. 

### 2.3. Synthesis of Pristine and Ag-TiO_2_ Nanomaterials 

TiO_2_ nanomaterials were synthesized by mixing 3 mL of titanium isopropoxide with 47 mL ethanol (50 mL) and fig leaf extract (10 mL) in a round-bottom flask and heating with stirring at 100 °C. Following homogenous mixing (30 min), diethylamine (10 mL) was added dropwise to that solution to obtain a white precipitate. Then, 50 mL of absolute ethanol was added to the reaction to make an azeotropic mixture. The entire reaction mixture was stirred at 100 °C for 24 h. After completing the reaction, the precipitant was separated using centrifugation. To eliminate contaminants from the precipitant, it was washed many times with distilled water and ethanol before being heated in an oven at 100 °C for 24 h to obtain the final TiO_2_ product. 

By following the same procedure as mentioned above, 50 mL of AgNO_3_ was added in different concentrations of 1%, 2%, 3%, and 4% *w/v* into the reaction mixture. The prepared Ag-doped TiO_2_ nanomaterials with four different concentrations were termed 1% Ag-TiO_2_, 2% Ag-TiO_2_, 3% Ag-TiO_2_, and 4% Ag-TiO_2_, respectively. Moreover, the prepared nanomaterials were calcined at 600 °C for four hours to get pure materials. 

DSSCs consist of several components, namely, a working electrode (semiconductor metal oxide), photosensitizer (dye), electrolyte, and counter electrode [23]. To obtain the final device, the sandwich shape is made by gathering the counter electrode (CE) and working electrode (WE) with the stacked dye; at the end, the electrolyte is injected in the middle. When sunlight hits the dye, the excited electron jumps from the highest occupied molecular orbital (HOMO) to the lowest occupied molecular orbital (LUMO). The free electron (e^−^) is injected from the LUMO of the dye to the conduction band (CB) of the semiconductor in the working electrode. The semiconductor (TiO_2_) transports the injected electron in the external electrical circuit, and electrons move to the counter electrode. Finally, due to the presence of electrolytes, a redox reaction takes place to regenerate the dye. The catalyzer’s role is to regenerate the oxidized electrolyte. 

### 2.4. Construction of Dye–Sensitized Solar Cells (DSSCs) Devices 

The fluorine-doped tin oxide (FTO) glasses were cleaned using soap, distilled water, and ethanol. The multimeter device was used to identify the conducting side of FTO glasses. A 0.25 cm × 0.25 cm area of FTO glasses was delimited by using Scotch tape around the edges of the glasses. Commercially available platinum (Pt) was used. The Pt thin film layer was deposited by the doctor blade method and then kept overnight at room temperature to dry. The Scotch tape was removed very carefully without any scratches. Finally, the counter electrode was heated in a furnace for 4 h at 450 °C. After that, it was kept overnight to cool at room temperature. 

As-prepared pristine and Ag-doped TiO_2_ were blended with distilled water, triton X-100, and acetylacetone in a conical flask and stirred for 24 h at 300 rpm to form a colloidal suspension. Ultrasonication was used to homogenize the TiO_2_ and Ag-doped TiO_2_ pastes for 30 min, respectively. The working electrode was prepared in the same way as the counter electrode. The working electrode was then immersed in a commercial di-tetra butyl ammonium cis-bis (iso thiocyanate) bis (2,2′-bipyridyl-4,4′ di carboxylato) ruthenium (II) (N719) dye solution overnight and kept in a dark place. After 24 h, the electrode was removed from the dye solution and washed several times with ethanol to remove the unsoaked dye.

A sandwich-shaped device was employed, with a counter electrode and a working electrode. As a result, the dye-coated electron transfer material layer faced the conductive side of the counter electrode. A spacer was placed on the working electrode as a three-sided gasket, leaving one side open for the injection of electrolyte. Heat was applied to the electrode to stick it and prevent leakage. Silver paste was applied to obtain higher electrical conductivity. The device was filled with a solution of an electrolyte (Iodolyte HI30-Solaronix, Aubonne, Switzerland) and sealed using Araldite. Both sides of the electrodes were connected through copper wire to measure the potential difference, and the device was put under a solar light simulator to measure its efficiency under a current density–voltage (J–V) characteristic. 

### 2.5. Characterization Techniques 

Pristine TiO_2_ and Ag-doped TiO_2_ nanomaterials were characterized using different methods. X-ray diffraction (XRD, Shimadzu Lab X-6000, Tokyo, Japan) with Cu-Kα radiation (λ = 1.5406 Å) was used to identify the peaks of synthesized nanomaterials, which had Bragg’s angles of between 20° and 80°. The surface morphologies of the prepared nanomaterials were investigated using field emission scanning electron microscopy (FESEM, JEOL JSM-7610F). Energy dispersed spectroscopy (EDS) was used to study the surface elemental analysis of the 2% Ag-doped TiO_2_ nanomaterial. The absorption spectra and optical properties of the prepared nanomaterials were observed using an ultraviolet–visible spectrophotometer (UV–vis, T80 PG instrument) in the wavelength range of 200 to 700 nm. A Fourier transform infrared spectrometer (FTIR, Thermo Scientific Nicolet iS10, Waltham, MA, USA) was used to study the infrared spectra at room temperature, ranging 500–4000 cm^−1^. The photovoltaic performance, including fill factor, efficiency, and current density–voltage (J–V) of fabricated devices, was measured using a Keithley 2400 source meter at 100 mW cm^−2^ of solar spectrum lighting.

## 3. Results and Discussion

### 3.1. X-ray Diffraction (XRD) Analysis

**Figure 1:**
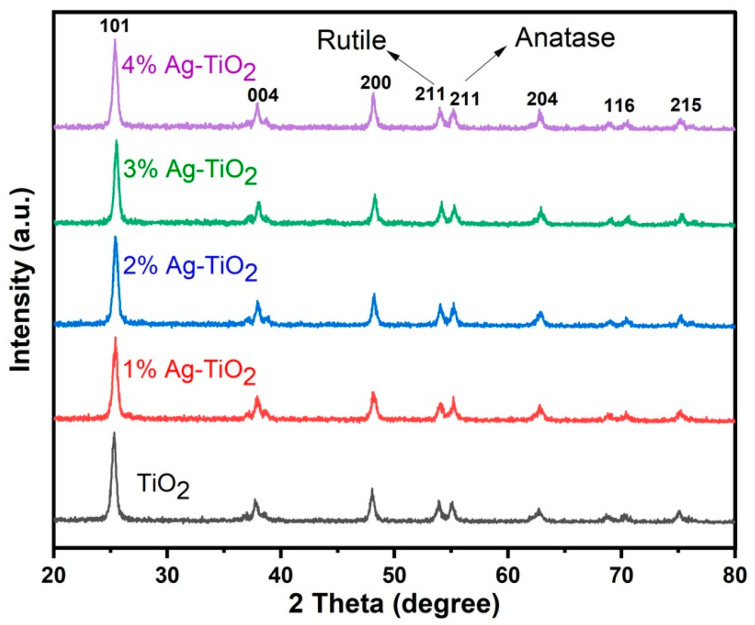
XRD pattern of pristine and Ag-doped TiO_2_ with 1, 2, 3, and 4% Ag.

The XRD pattern of pristine and Ag-doped TiO_2_ is shown in Figure 1. The peaks of pristine and Ag-doped TiO_2_ exhibit both anatase and rutile phases [11,16] at 2θ = 25.4, 37.7, 48.1, 54.0, 55.0, 62.2, 70.4, and 75.1 degrees, which correspond to (101), (004), (200), (100), (211), (204), (116), and (215) crystal planes, respectively. The tetragonal form of rutile and anatase is confirmed by corresponding to cards No. 21-1272 and No. 21-1276, respectively [16], and no impurity peaks in the pattern were detected. Ding et al. reported that mixed phases of TiO_2_ are good for DSSCs, and this might be due to more dye adsorption and improved electron transportation [24]. The crystallite size ranges from 12.64 to 13.47 nm, which was calculated by the following Scherrer equation with the highest intensity peak (101).

$$D=\frac{K\lambda }{\beta cos\theta }$$

Here, *K* is a constant with a value of 0.9, λ is the wavelength of CuKα radiation (1.540 Å), *θ* is Bragg’s angle, and *β* is the full width at half maximum. There is a slight variation in the crystallite size due to the phase composition of rutile and anatase. The XRD patterns of Ag-doped TiO_2_ nanomaterials did not exhibit any additional silver peaks because the silver particles dispersed on the surface of TiO_2_ and a small amount of Ag could not be detected by XRD analysis. Furthermore, Figure 1 revealed that the doping of Ag did not affect the mixed phase of TiO_2_ [17,18]. FE-SEM images clearly show the silver particles dispersed on the surface of TiO_2_.

By using the following equation, the lattice parameters of the tetragonal phase were determined:

$$\frac{1}{a2}=\frac{h2+k2}{a2}+\frac{l2}{c2}$$

where a and c are lattice parameters; h, k, and l are Miller indices; and d is interplanar spacing. The study reported an inverse association between the lattice parameter and Bragg’s angle, which is clear from the calculated lattice parameter as mentioned in Table 1 [25].

The calculated data showed that by increasing the content of Ag (1, 2, and 3%), the lattice parameters decreased slightly, which corresponds to strong diffraction peaks. On the other hand, the lattice parameters increased slightly at 4% Ag content because of the peak position shifted to a lower angle.

The smaller crystallite size has a higher surface area, which affects the dye loading as well as the performance of DSSCs. Based on previous studies, it is noted that crystallite size is mostly related to the performance of the solar cell [15,16]. In this study, however, it was found that the highest PCE was achieved with a 13 nm crystallite size by using 4% Ag dopant, and the expectation was a high injection time, which might be due to more Ag particles available on the TiO_2_ surface, which enhanced the PCE [26].

The dislocation density (*δ*) and microstrain (*ε*) were calculated using the following equations:

δ=1D^2

where D is the crystallite size of nanomaterials. Dislocation density is the length of line dislocation inside the crystallite per unit, and it is responsible for the hardness of nanomaterial crystals. Mustapha et al. reported that dislocation density increases by decreasing the crystallite size of nanomaterials [27]. Therefore, the prepared nanomaterials are harder than bulk TiO_2_.

$$\varepsilon =\frac{\beta }{4\tan\theta }$$

where β is full width at half maxima, and θ is Bragg’s angle. It is observed from Table 1 that the dislocation density and microstrain have opposite correlations with the crystallite size of nanomaterials.

### 3.2. Fourier Transform Infra-Red (FT-IR) Analysis

**Figure 2:**
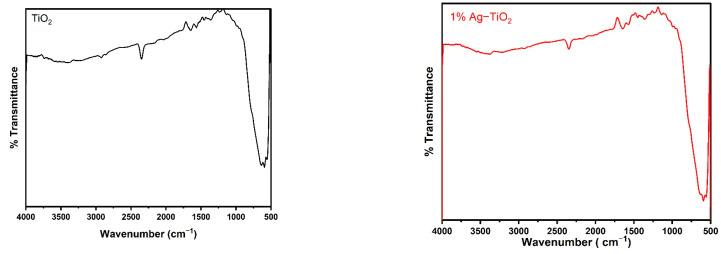

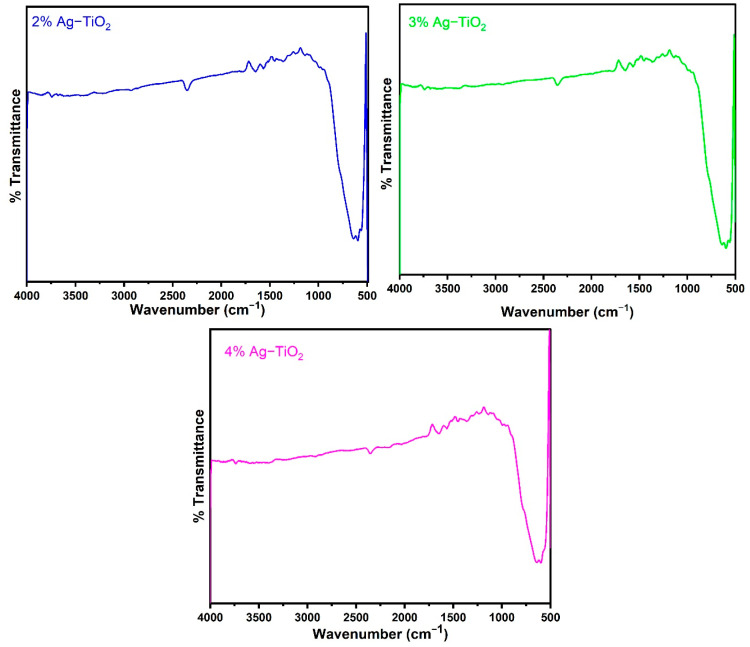
FTIR spectra of pristine TiO_2_ and TiO_2_ nanomaterials doped with 1, 2, 3, and 4% Ag.

Figure 2 displays the FT-IR spectra of the pristine and Ag-doped TiO_2_ nanomaterials with 600 °C annealing in the 500–4000 cm^−1^ range. The FT-IR spectroscopic studies were carried out to confirm the presence of functional groups in the synthesized pristine and Ag-doped TiO_2_ nanomaterials. The obtained bands between 1639 and 1659 cm^−1^ are assigned to bending modes of the -OH groups due to the adsorbed water molecules on the surface [24,25]. It has been observed that pristine and doped TiO_2_ with different concentrations of Ag reveal almost similar spectra. The presence of an O-Ti-O lattice is indicated by the strong absorption bands in the 583–634 cm^−1^ region, which confirm the formation of Ti-O stretching bands [18]. Moreover, the peak around 586 cm^−1^ slightly increases the wavenumbers with increasing concentrations of Ag-doping in TiO_2_, as shown in Figure 2, which suggests that Ag interacts with TiO_2_ [26].

### 3.3. Field Emission Scanning Electronic Microscopy (FE-SEM)

**Figure 3:**
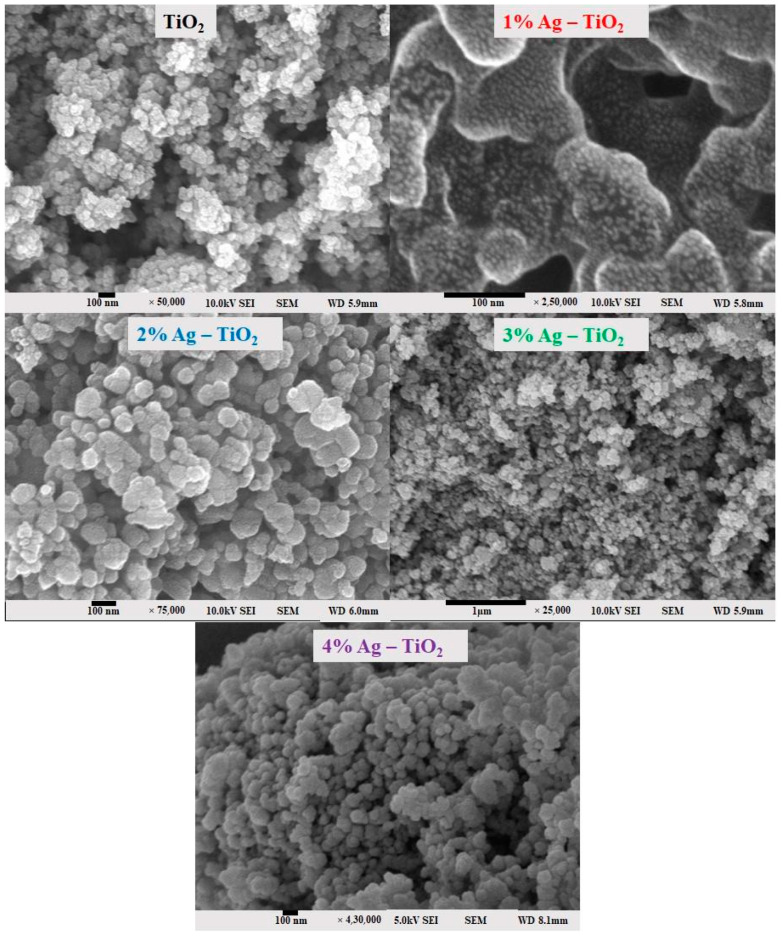
FE-SEM surface morphology analysis of pristine TiO_2_ and TiO_2_ nanomaterials doped with 1, 2, 3, and 4% Ag.

The surface morphology of pristine TiO_2_ and Ag-doped TiO_2_ with 1, 2, 3, and 4% Ag was investigated using field emission scanning electron microscopy (FE-SEM) and is shown in Figure 3. The FE-SEM images clearly confirm the silver particles dispersed on the surface of TiO_2_. It is possible that it tends to form aggregates, which may be due to the presence of van der Waals forces between the nanoparticles [6]. The nanomaterials exhibited a spherical morphology and aggregated together. The 4% Ag-doped TiO_2_ nanomaterials had quite a smooth surface and low agglomeration.

**Figure 4:**
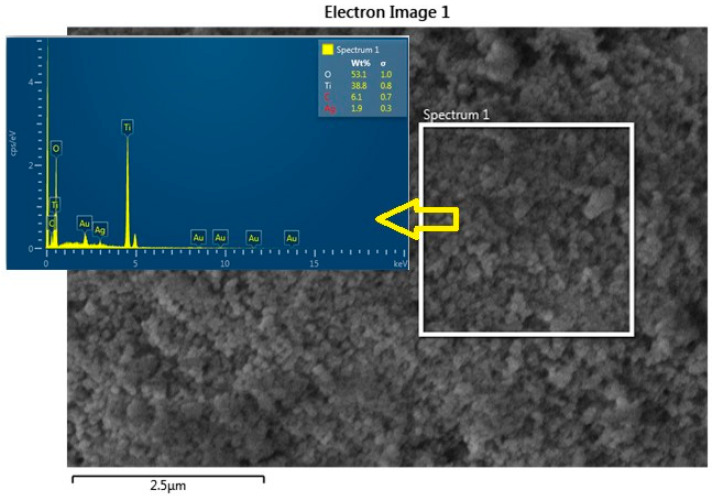
EDS analysis of the 2% Ag-doped TiO_2_ nanomaterial.

**Figure 5:**
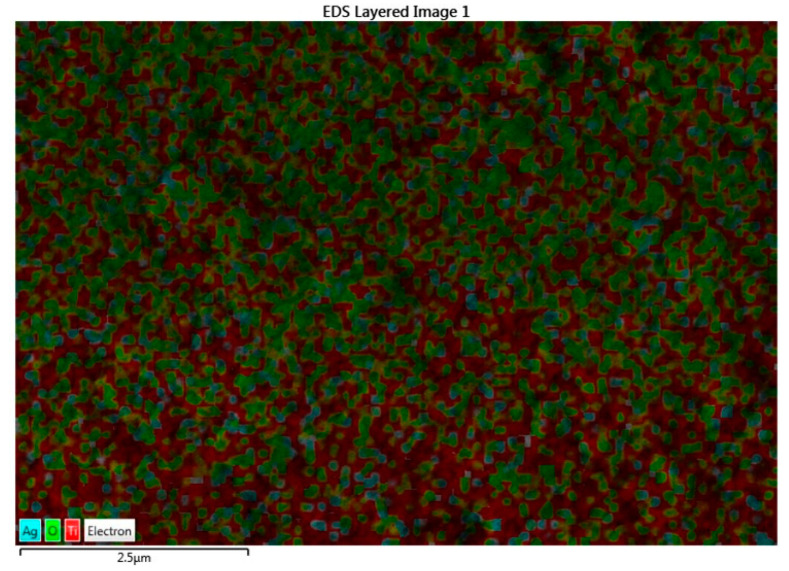
EDS image of the 2% Ag-doped TiO_2_ nanomaterial.

**Figure 6:**
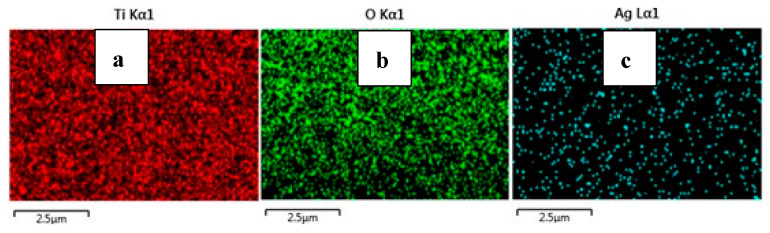
EDS images of (**a**) Ti, (**b**) O, and (**c**) Ag.

From the FE-SEM figure, however, it can be ruled out that Ag nanoparticles were observed on the surface of TiO_2_ due to the increase in the content of silver, which led to enhanced dye loading. Moreover, the particle size was calculated using Image J 1.54d software, and the results revealed that size varies with the concentration of Ag doping, which is in good agreement with the XRD results. The surface elemental characterization of 2% Ag-TiO_2_ nanomaterial was analyzed using EDS. To confirm the presence of elements, the EDS analysis was performed at various selected areas from the FE-SEM image. It is clear from Figure 4, Figure 5 and Figure 6 that all elements such as Ti, O, and Ag were present in the prepared nanomaterials, which endorsed the doping of Ag.

### 3.4. Optical Properties

**Figure 7:**
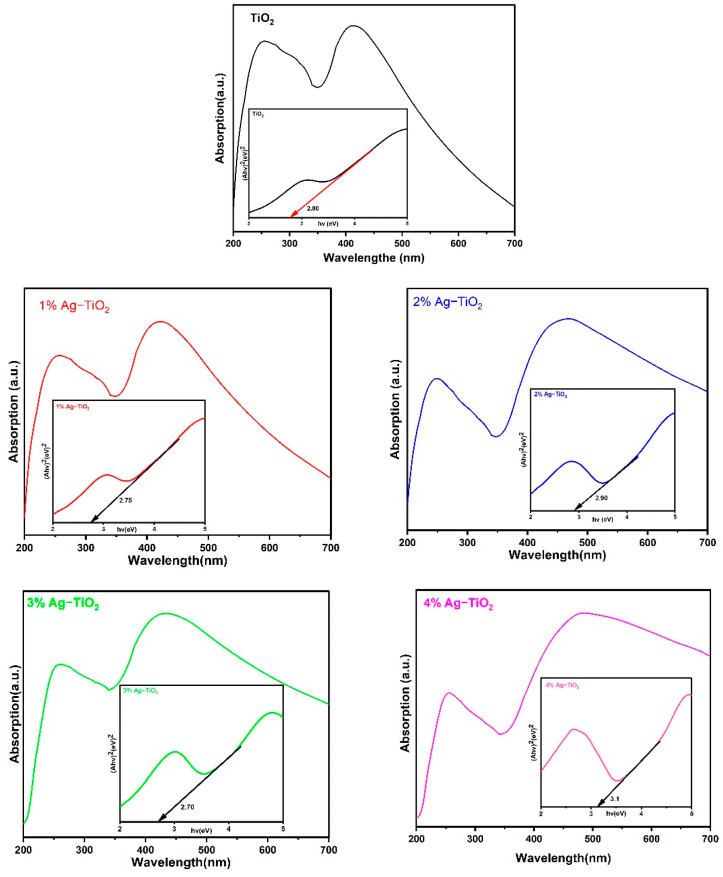
UV–Vis spectra of pristine TiO_2_ nanomaterial with an optical band gap.

The absorption spectra and energy band gap of pristine and Ag-doped TiO_2_ nanomaterials were studied using UV–vis spectroscopic analysis. The optical properties were demonstrated by the UV–vis absorption spectra of synthesized materials in the 200–700 nm wavelength ranges. The absorption spectrum of pristine TiO_2_ has a strong absorption band at around 255 and 418 nm. After doping Ag ions (1% to 2%), a corresponding red shift of the absorption edges toward a longer wavelength (around 420–481 nm) with respect to pristine TiO_2_ was observed in Figure 7. The absorbance of Ag-TiO_2_ nanomaterials rises as the Ag doping level increases, and this might be attributed to surface plasmon resonance (SPR) of nanoparticles or electromagnetic field interference with the conduction electrons of Ag particles dispersed over the TiO_2_ matrix [15]. By using Tauc’s formula, the optical band gap energy of the prepared nanomaterials can be calculated as given below [24]:

(αhν)^n^ = A(hν − E_g_)

where A represents a material-related constant, E_g_ represents the optical band gap, hν is the photon energy, α is the absorption coefficient, and n is determined by the nature of the transition (n = ½, and 2 for indirect and direct band gaps, respectively).

As shown in Figure 7, to obtain the direct E_g_ values from these spectra, the linear portion of the Tauc curve was extrapolated between hν on the X-axis and (αhν)^2^ on the Y–axis. The optical band energy has been found to be 2.80 eV for pristine TiO_2_, whereas the calculated band gap first decreased to 2.75 and 2.70 eV with 1% and 3% Ag, respectively, and then increased to 2.90 and 3.10 eV with 2% and 4% Ag, respectively. The SPR, which involves the interaction of an electromagnetic field with electrons of Ag nanoparticles dispersed on the surface of TiO_2_, is responsible for the higher energy band gap caused by increases in concentration with 4% of Ag. The results suggest that the DSSC device’s performance can be enhanced by using 2 and 4% Ag, which leads to increased light absorption in the visible region [15].

### 3.5. Photovoltaic Performance of Device

**Figure 8:**
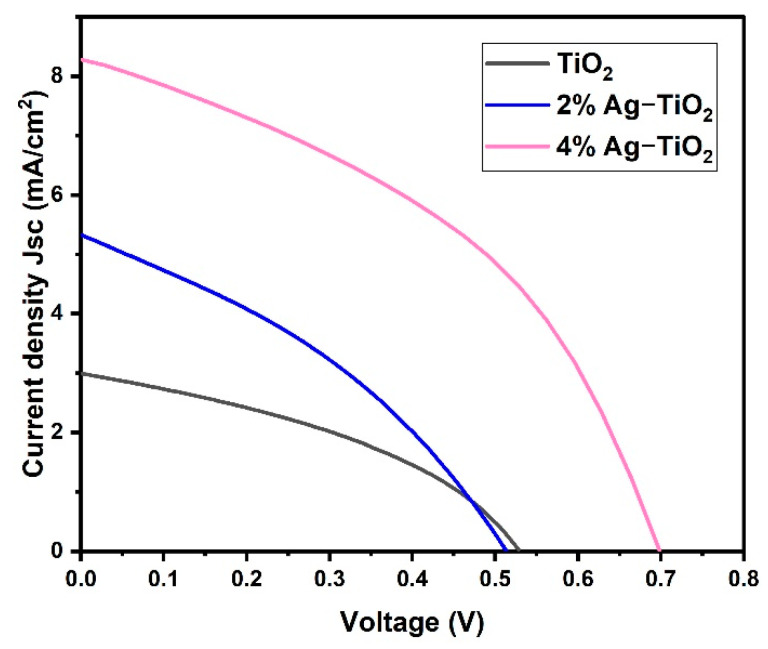
Current density vs. voltage of pristine TiO_2_ and TiO_2_ doped with 2% Ag and 4% Ag.

Among the five prepared nanomaterials, only three samples (pristine TiO_2_ and 2% and 4% Ag-doped TiO_2_) exhibited good photovoltaic performance. However, the J–V characteristics of the DSSCs devices are shown in Figure 8 using studied pristine TiO_2_ and doped TiO_2_ with Ag percentages as photoanodes. Herein, the effect of Ag doping on the photovoltaic performance of fabricated cells such as pristine TiO_2_, 2% Ag-TiO_2_, and 4% Ag-TiO_2_ films was estimated in contradiction to the Pt counter electrode, I^−^/I^3−^ redox electrolyte, and N719 dyes under an illumination intensity of 1 sun. Additionally, the photovoltaic parameters are summarized in Table 2.

The power conversion efficiency (PCE) of DSSCs is calculated by the following equation [28].

$$\mathrm{PCE}=\;\frac{Voc\times Jsc\times FF}{\mathrm{Pin}}\times 100\%\;$$

where Pin is the intensity of the incident light, *V_oc_* is the open circuit voltage, *J_sc_* is the current density, and FF refers to the fill factor.

The pristine TiO_2_ cell exhibited an open circuit voltage (V_oc_) of 533 mV, a short circuit current density (J_sc_) of 2.992 mA/cm^2^ and a fill factor (FF) of 0.388 with a light power illumination (P_in_) of 100 mW/cm^2^, which corresponds to an overall power conversion efficiency (PCE) of 0.62%. The PCE was increased to 2.45% in the case of 4%Ag-TiO_2_, which exhibits a J_sc_ of 8.336 mA/cm^2^, V_oc_ of 698 mV, and an FF of 0.422. The J–V curve indicates that Ag ion implantation has a positive impact on the photoelectric performance of photoanodes. Moreover, J_SC_ demonstrates a clear upward trend with rising Ag ion concentrations. The higher efficiency of 4%Ag-TiO_2_ is probably due to the interfacial charge transfer process through Ag. Additionally, this might be attributed to the high energy band gap, high adsorption of dye on the TiO_2_ surface, high charge transport, and minimizing charge recombination [28]. The lower efficiency of pristine TiO_2_- and 2% Ag-TiO_2_-based DSSC devices is attributed to the lower energy bandgap and low dye loading compared to 4% Ag-TiO_2_-based DSSCs. The results revealed that the photo-conversion efficiency of DSSCs has been increased by surface engineering of the photo-anode materials.

## 4. Conclusions

In conclusion, the facile green modified solvothermal method was successfully used to prepare pristine TiO_2_ and Ag-doped TiO_2_ with 1, 2, 3, and 4% of Ag. The influence of the deposition of Ag onto the TiO_2_ photoanode nanomaterial on the performance of DSSCs was investigated. EDS analysis confirmed the formation of Ag-doped TiO_2_. In addition, the formation of mixed rutile and anatase phases was confirmed by the XRD pattern. According to J–V curves, Ag ions showed an excellent impact on the current density and efficiency of devices. Moreover, Ag ion incorporation into TiO_2_ at a high concentration (4%) enhanced dye adsorption and reduced charge-transfer resistance because the deposited Ag ions served as mediators for electron transport. Therefore, the photocurrent performance of fabricated DSSCs using 4% Ag-TiO_2_ showed a better power conversion efficiency of 2.45%.

## Figures and Tables

**Table 1:** Lattice parameters of tetragonal form and the crystallite size, dislocation density, and micro-strain of pristine and Ag-doped TiO_2_ (1% to 4%) with 101 Miller indices.

Doping of Ag at %	2 Theta (deg)	FWHM β (deg)	Crystallite Size, D (nm)	Lattice Parameters (nm)		c/a	Interplanar Spacing, d (nm)	Density	Micro Strain ε
				a	C				
TiO_2_	25.29	0.61035	13.3	0.4973	0.4447	0.89	0.3516	0.0056	0.6799
1% Ag-TiO_2_	25.40	0.64377	12.6	0.4952	0.4429	0.89	0.3501	0.0062	0.7139
2% Ag-TiO_2_	25.45	0.63237	12.8	0.4943	0.4421	0.89	0.3495	0.0060	0.7000
3% Ag-TiO_2_	25.52	0.60451	13.4	0.4930	0.4409	0.89	0.3486	0.0055	0.6673
4% Ag-TiO_2_	25.40	0.62208	13.0	0.4952	0.4429	0.89	0.3501	0.0058	0.6899

**Table 2:** Performance of devices based on pristine TiO_2_ and TiO_2_ doped with 2% Ag and 4% Ag.

Sample Code	V_oc_ (mV)	J_sc_ (mA/cm^2^)	FF	PCE%
TiO_2_	533	2.992	0.388	0.62
2% Ag–TiO_2_	513	5.324	0.354	0.97
4% Ag–TiO_2_	698	8.336	0.422	2.45

## Data Availability

Not applicable.
